# Evolution of conductive filament and its impact on reliability issues in oxide-electrolyte based resistive random access memory

**DOI:** 10.1038/srep07764

**Published:** 2015-01-14

**Authors:** Hangbing Lv, Xiaoxin Xu, Hongtao Liu, Ruoyu Liu, Qi Liu, Writam Banerjee, Haitao Sun, Shibing Long, Ling Li, Ming Liu

**Affiliations:** 1 Lab of Nano-fabrication and Novel Devices Integration, Institute of Microelectronics, Chinese Academy of Sciences, Beijing 100029, China

## Abstract

The electrochemical metallization cell, also referred to as conductive bridge random access memory, is considered to be a promising candidate or complementary component to the traditional charge based memory. As such, it is receiving additional focus to accelerate the commercialization process. To create a successful mass product, reliability issues must first be rigorously solved. In-depth understanding of the failure behavior of the ECM is essential for performance optimization. Here, we reveal the degradation of high resistance state behaves as the majority cases of the endurance failure of the HfO_2_ electrolyte based ECM cell. High resolution transmission electron microscopy was used to characterize the change in filament nature after repetitive switching cycles. The result showed that Cu accumulation inside the filament played a dominant role in switching failure, which was further supported by measuring the retention of cycle dependent high resistance state and low resistance state. The clarified physical picture of filament evolution provides a basic understanding of the mechanisms of endurance and retention failure, and the relationship between them. Based on these results, applicable approaches for performance optimization can be implicatively developed, ranging from material tailoring to structure engineering and algorithm design.

The phenomenon of resistance switching, which occurs in functional materials stimulated by electrical fields, magnetic fields, temperature or illumination, can be applied in diverse fields^1^,^2^,^3^. Typically, redox-based resistive switching memory (RRAM) is regarded as the preliminary candidate for the next generation of non-volatile memory, considering its outstanding characteristics such as low power, fast switching, high endurance and ultimate atomic scaling^4^,^5^,^6^,^7^,^8^,^9^. As the popular use of Cu in nowadays damascene interconnects, the Cu based electrochemical metallization cells (ECM) are of particular importance and more competitive compared with other types emerging memory, by considering the compatibility and fabrication cost. In the early years, chalcogenides such as sulphides^10^, iodides^11^, selenides^12^ and tellurides^13^, were used as ECM switching materials. These materials showed very promising performance except the ultra-low switching voltage, which allowed small thermal and electrical noises to disturb the resistance state. In other words, these material systems suffered from unfavorable data retention^12^. Recently, the use of oxide materials as electrolytes was explored, due to their relative low ionic mobility^14^. The switching voltages of these devices were successfully increased to the levels of complementary metal oxide semiconductor (CMOS) operating voltages and data retention was greatly improved. However, oxide-based ECM had reduced endurance. A single TaO_x_ device (Cu/TaO_x_/Pt structure) lasted 10^4^ cycles^14^ and a bilayer CuTe/GdO_x_ device achieved 10^8^ cycles^15^, which was much smaller than the 10^10^ cycles achieved by GeSe^12^. Recently, high resolution transmission electron microscopy (HRTEM) has been used to characterize nano-scaled conductive filaments and their formation and rupture dynamics, significantly aiding our understanding of the switching mechanism^16^,^17^,^18^. However, very few studies have addressed the topic of filament evolution under continuous programming, which is of high importance in settling the reliability issue. Questions regarding changes in filament morphology or composition over different switching cycles and the relationship between retention and endurance have seldom been addressed.

We designed a 1 k-bit array for an HfO_2_-based ECM device with a one-transistor-one-resistor (1T1R) structure and systematically investigated the failure and cycled retention loss in them. By tracing the evolution trends of resistance state during the repetitive switching cycles, high resistance state (HRS) degradation was found to exist in the majority of cases of endurance failure. HRTEM characterization revealed that the copper concentration inside the filament increased in the failed device, whereas the filament size was slightly affected. This finding was further supported by cycle dependent HRS and low resistance state (LRS) retention measurements. HRS retention decreased with cycling, whereas LRS retention was improved. The results presented here provide a basic understanding of the mechanisms of endurance and retention failure and the relationships between them.

## Results

### Sample configuration and the basic operation of bipolar resistive switching

**Figure 1:**
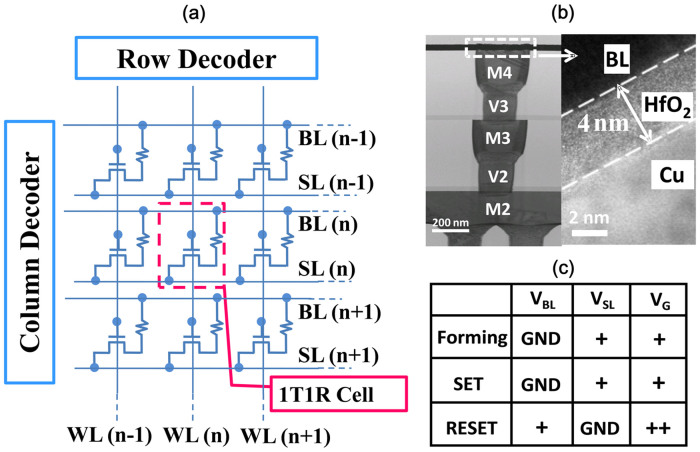
The HfO_2_ based 1T1R ECM device and array used in this study. (a) The schematic of the 32 × 32 1T1R array. The gates of the regular arranged transistors were connected by a wordline in low and the top electrodes of the ECM cells were linked by a bitline in column. (b) The cross-section TEM image of 1T1R structure. The transistor was fabricated by a 0.13 μm CMOS process with a Cu/HfO_2_/Pt RRAM device on top of it. The Cu plug served as the bottom electrode. The thickness of the HfO_2_ layer was about 4 nm. (c) The test conditions of the ECM cell.

A Cu/HfO_2_/Pt structure was integrated on the drain of transistor to build a 1T1R structure. The Cu plug of the top metal, after chemical-mechanical planarization, served as the bottom electrode. The gates of the regularly-arranged transistors were connected by a wordline (WL) in row and the top electrodes (TE) of the ECM cells were linked by a bitline (BL) in column. More detailed information can be found in the experimental section and in Supplementary Figures S1a to S1f. The schematic diagram and the optical image of the 1 kb 1T1R array are shown in Figure 1a. Figure 1b shows the TEM images of the device cross-section. The thickness of the HfO_2_ layer was approximately 4 nm. The Cu/HfO_2_/Pt device exhibited bipolar switching behavior. A positive voltage bias was applied on bottom electrode (BE) for the forming and SET operation (from HRS to LRS), whereas for the RESET operation (from LRS to HRS), the TE was positively biased. In the case of the 1T1R structure used here, the forming/SET and RESET were programmed by the source line and bitline, respectively. The test conditions are summarized Figure 1c.

### Endurance failure behavior and the HRS/LRS conduction mechanism

**Figure 2:**
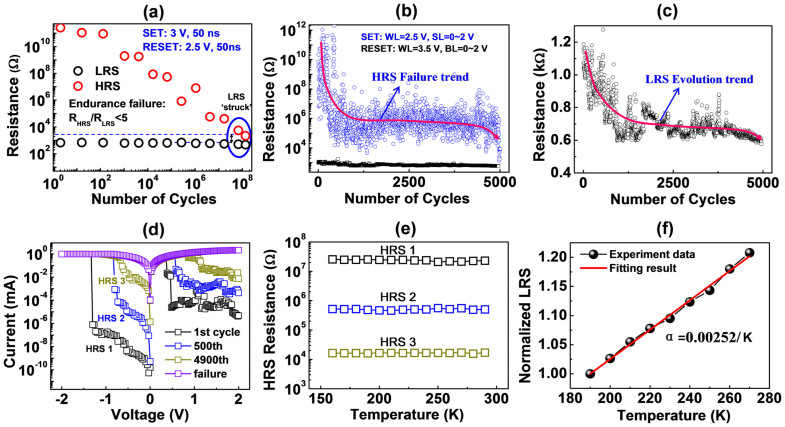
The evolution of the endurance failure and HRS/LRS conduction mechanism. (a) The pulse endurance test of the 1T1R cell with a SET pulse with 3 V/50 ns and a RESET pulse with 2.4 V/50 ns at room temperature. The resistance states were intermittently measured with a small read voltage of 0.1 V. The HRS was found to degrade with cycling, whereas the LRS remained nearly constant. (b) The DC switching test of the 1T1R cell, showing almost the same trend as the pulse test. (c) The enlarged LRS change over cycles of (b). (d) The linear plot of I–V curves after the 1^st^, 500^th^ and 4900^th^ cycles. The SET voltage decreased and the RESET voltage increased with cycling. (e) The relationship between the temperature and HRS of different resistance. The HRS was nearly independent of temperature, indicating that the dominant conduction in the HRS was tunneling. (f) The temperature dependence of the LRS.

Figure 2a shows the evolution trends of the HRS and LRS of the 1T1R cell under repetitive switching cycles by voltage pulses at room temperature with the SET pulse and RESET pulse at 3 V/50 ns and 2.4 V/50 ns, respectively. The resistance states were intermittently measured with a small read voltage of 0.1 V. The criterion for switching failure was defined as R_HRS_/R_LRS_ < 5. It is found that the HRS was reduced with cycling, whereas the LRS showed little change. The shrinkage of the on/off ratio (R_HRS_/R_LRS_) was caused mainly by the degradation of the HRS. Almost all of the devices are finally ‘stuck’ at the LRS, indicating that this is a common failure behavior for this ECM cell. A similar phenomenon has also been observed in WO_3_ and GeSe systems^19^,^20^. In a few cases, the cells failed at ultra-high resistances above 10^13^ Ω, resulting from the physical damage of the Cu plug by the high current density. These failures should be separated from the intrinsic switching failure of the ECM cell.

The DC switching cycle provided more detailed information about changes in resistance and switching voltage. As shown in Figure 2b, the failure behavior during DC switching was almost the same as that of pulse testing. During the first few hundred cycles, the HRS was highly insulated. It then degraded to 10^4^ ~ 10^6^ Ω and finally to approximately 1 kΩ. The LRS change over cycles is shown in Figure 2c. A slight decrease in LRS from 1.2 kΩ at initial cycles to 0.6 kΩ can be observed. The V_SET_ decreased with cycling, whereas the V_RESET_ increased, as shown in the semi-log plots of I-V curves after the 1^st^, 500^th^ and 4900^th^ cycles (Figure 2d). More obvious trends in V_SET_ and V_RESET_ changes with cycle can be found in Supplementary Figures S2a and S2b. The increase in V_RESET_ suggested that the retraction of filament became more difficult with cycling.

To elucidate the physical nature of switching failure, we first need to clarify the transport mechanism of the HRS and LRS. Many conduction models have been suggested to describe electron transport in the switching layer, including tunneling^21^, Schottky emission^22^, space charge current^23^ and F-P emission^24^. An effective way to distinguish between these models is to measure temperature dependence. Figure 2e shows the relationship between the temperature and HRS at different resistance states. The HRS was found to be nearly independent of the temperature, indicating that tunneling was the dominant form of conduction in the HRS. This result is consistent with the tunneling gap model proposed by Menzel *et al.* to account for the multi-level storage of a Cu-SiO_2_ device^21^ and the trap assist tunneling model by Yu *et al.* to describe the temperature insensitivity of the HRS in HfO_2_-based RRAM^25^. The HRS resistance is mainly determined by the tunneling gap between the filament tip and the counter electrode. The HRS resistance degraded with cycling, meaning that the corresponding tunnel gap length was reduced, as can be clearly seen from the result of the tunneling model calculations shown in Supplementary Figure S3. The LRS had a high linear I–V curve with positive temperature dependence (Figure 2f), indicating the metallic property of the LRS.

### HRTEM analysis of the filament evolutions after repetitive switching cycles

**Figure 3:**
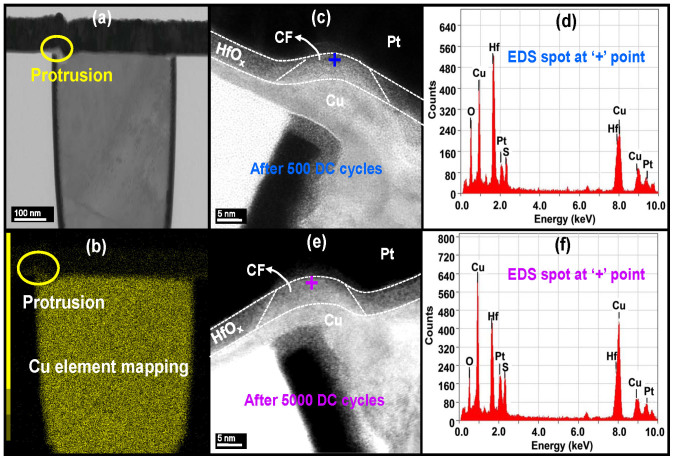
TEM analysis of the filament regions for the samples with different switching cycles. (a) TEM image of the cross-section of the cycled device. A small depression can be seen at the left corner of the copper plug, resulting in a protrusion-like shape therein. (b) The copper element mapping of (a). (c) The magnification of the filament region after 500 DC cycles. The filament was located on the corner of the copper plug due to the enhancement of the local electric field. (d) EDS analysis of the spot of the filament region after 500 DC cycles. (e) The magnification of the filament region after 5000 DC cycles. This device failed at the LRS. Slight increase of the filament size was found. (f) The EDS analysis of the spots of the filament regions after 5000 DC cycles. The Cu signals after 5000 cycles were greatly enhanced from the Cu signals after 500 cycles, indicating that Cu accumulated in the filament region during cycling.

The evolution of the filament morphology and composition after various switching cycles were characterized by HRTEM. Two LRS samples were operated by 500 and 5000 DC cycles under the programming conditions shown in Figure 2(b), then prepared for TEM analysis. The cross-section of the cycled device is shown in Figure 3a. A small depression can be seen in the left corner of the copper plug, resulting in a protrusion-like shape, as shown by the electron energy loss spectroscopy (EELS) of the copper element mapping in Figure 3b. The filament created during the SET operation was likely to be located on this protrusion, due to the local enhancement of electrical field^24^. Figure 3c shows a magnification of the left plug corner of the 500-cycled sample. A cone-like area with low contrast can be observed in the HfO_2_ layer, located on the top of the protrusion. An electron energy-dispersive spectroscopy (EDS) analysis, taken near the Pt electrode to minimize the copper signal from the bottom electrode, shown in Figure 3d, revealed that this low contrast area contained a large amount of copper, corresponding to the localized conductive region of the LRS device. The cone shape of the filament was consistent with previous TEM observations and the *in-situ* TEM measurement on filament growth and rupture dynamic of the Ag/ZrO_2_/Pt device^17^,^18^,^26^, suggesting that the filament grew from the copper electrode toward the Pt electrode and the resistance switching took place at the interface between the filament and inert electrode. The neck size of the filament near the Pt electrode was about 15 nm. Figure 3e shows the magnification of the filament region in the 5000 cycled sample. A similar cone-shaped filament was observed in this device, with a similar size of around 16 nm. Although more copper in the region out of the so-called cone shape filament could be found, the most remarkable difference between these two samples was the concentration of copper inside the filament. As shown in Figure 3f, the copper peaks of the EDS signal were more enhanced in the failed device. The TEM results revealed that the copper concentration in the filament increased with the switching cycles, possibly causing the filament to rupture and leading eventually to RESET failure.

### Cycle-dependent HRS and LRS retention measurements

**Figure 4:**
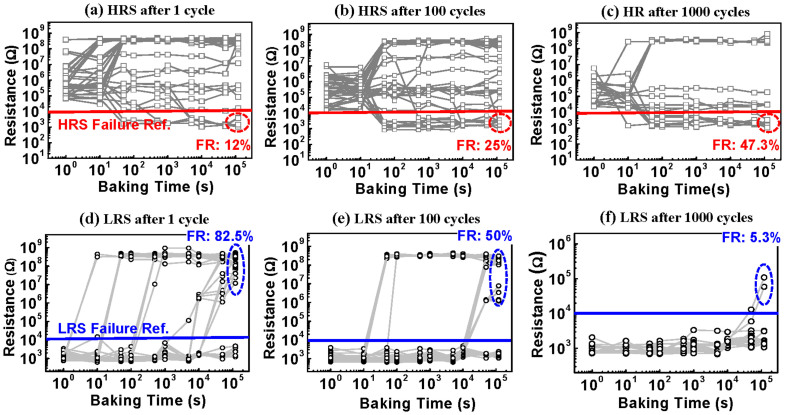
The retention test of the HRS and LRS after various switching cycles. The HRS retention after (a) 1 cycle, (b) 100 cycles and (c) 1000 cycles, with a baking temperature of 180°C. The HRS either changed to the LRS or moved to higher resistance states. The probabilities of changing to the LRS were 12% at 1 cycle, 25% at 100 cycles and 47.3% at 1000 cycles. As the switching cycles increased, the HRS states were more likely to change to the LRS, implying that HRS retention was degraded with cycling. The LRS retention after (d) 1 cycle, (e) 100 cycles and (f) 1000 cycles with a baking temperature of 180°C. The LRS retention was greatly improved with the cycling. After 1000 cycles, the failure rate was reduced from 82.5% to 5.3%.

The dependence of the retention characteristics on the switching cycles provided another clue about the evolution of filament properties. Six groups of devices, each containing 50 cells, were programmed to the HRS and LRS after 1, 100, and 1000 DC switching cycles, then baked at 180°C in an oven. The resistance states of these devices were checked periodically with a read voltage of 0.1 V when cooled down to room temperature. A reference resistance of 10^4^ Ω was defined to judge the HRS or LRS retention failure. The HRS of the devices either changed to the LRS or moved to higher resistance states, as shown in Figures 4a to 4c. The probabilities of changing to the LRS were 12% at 1 cycle, 25% at 100 cycles and 47.3% at 1000 cycles. The HRS retention therefore reduced with cycling. The failure of HRS retention can be interpreted as gap filling by vertical diffusion of copper from the residual filament, leading to the re-connection of the tunnel gap as a result. The degradation of HRS retention with cycling stemmed from a reduced gap length, as shown in Figures 2a and 2b, and an increase in the copper concentration in the filament. Narrowing of the tunnel gap means that less copper dopant was required to reconnect it and the increased copper concentration in the filament means a higher gradient for copper diffusion, both of which resulted in easier reconnection of the tunnel gap. Figures 4d to 4f show the LRS retention after 1, 100 and 1000 DC cycles. Two pieces of information can be obtained from the cycling-dependent LRS retention. First, the LRS retention improved with cycling. Compared with the 1 cycle group, the failure rate of the 1000 cycle group was reduced from 82.5% to 5.3%. Second, for the 1 cycled and 100 cycled groups, abrupt changes from the LRS to HRS were observed. The LRS retention failure was related to lateral diffusion of Cu from the filament to its surroundings. The improved LRS retention with cycling can be easily explained by the increased copper concentration or size enlargement of the filament due to the accumulation effect, requiring more time or a higher temperature for depletion. The gradual increases in the LRS resistance of the 1000 cycle group (Figure 4f) correlated to the shrinkage of the filament radius caused by lateral diffusion. With lower copper concentrations or smaller filament size, as in Figures 4d and 4e, the abrupt change can be resulted from the cutting off of conducting path by copper loss. The cycling-dependent retention characteristics of the HRS and LRS were in good accordance with the Cu accumulation mechanism.

## Discussion

**Figure 5:**
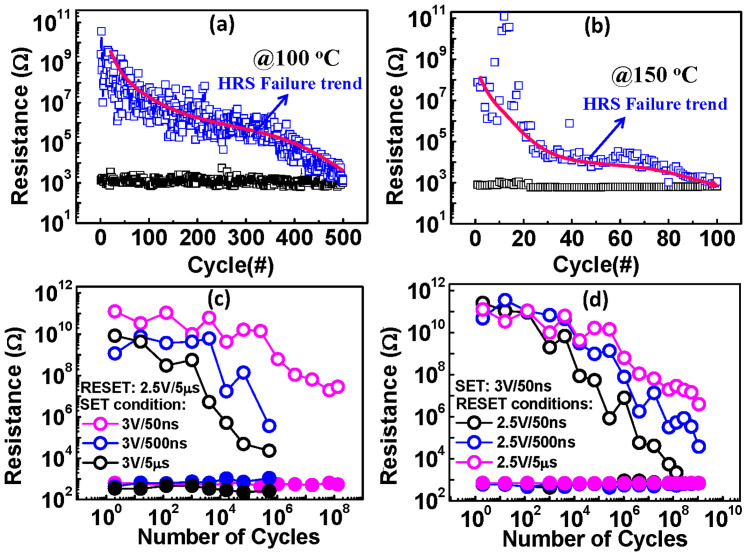
The dependence of the operation temperature and pulse condition on the endurance. (a) The high temperature DC endurance test at 100°C. (b) The high temperature DC endurance test at 150°C. (c) The endurance test results with varied SET pulse width and fixed RESET pulse. (d) The endurance test results with fixed SET pulse and varied RESET pulse width.

Based on the above analysis, we can draw a physical image of filament evolution with cycling. The motion of copper dopants from the copper electrode into the electrolyte contributed to the formation of copper rich filaments^27^. At the initial few cycles, the filament contained relatively less copper. A lower RESET voltage and worse LRS retention were thus observed. As the switching cycle continued, the copper concentration in the filament increased, making filament rupture more difficult. At this stage, a higher RESET voltage and improved LRS retention were exhibited. The degraded HRS resistance was caused by the reduced tunnel gap during RESET. The copper accumulation was accelerated by high temperature programming. As shown in Figures 5a and 5b, DC endurance at 100°C and 150°C were reduced to about 500 cycles and 100 cycles, respectively, compared with 5000 DC cycles under room temperature operation as shown in Figure 2b. Copper accumulation inside the electrolyte was related to the flux difference of copper species motion forward and backward across the electrolyte surface during the SET and RESET operations. Although the increase in copper in the electrolyte cannot be considered to be equivalent to the copper accumulation inside the filament, a proportional relationship between them can be expected. Based on this relationship, we predict that a shorter SET pulse, longer RESET pulse and lower SET compliance current would be favorable for improving endurance performance. With a shorter SET pulse and lower compliance, fewer copper ions would flow into the electrolyte, and for a longer RESET pulse, more copper would retract back into the electrode, which would help to alleviate copper accumulation during cycling. The endurance test results in Figure 5c (varied SET pulse width/fixed RESET pulse) and Figure 5d (fixed SET pulse/varied RESET pulse width) verified the hypothesis well. More than 10^9^ endurance was achieved by tailoring the proper SET and RESET pulse conditions. However, a bi-layer structure or the addition of a buffer layer between the electrolyte layer and active electrode may adjust the hopping barriers of copper species into and out of the electrolyte, which would improve the endurance characteristics. The performance of the TiO_2_/Ta_2_O_5_ device was found to be superior to the single Ta_2_O_5_ device^15^,^28^. The recently reported high performance ECM test chips all have bi-layer structures^15^,^29^,^30^.

In summary, the failure mechanisms of endurance and retention of an HfO_2_-based ECM were studied in a 1 kb array. The shrinkage of the on/off ratio was largely caused by the degradation of the HRS. Based on an HRTEM analysis, the copper accumulation in the filament was found to be the dominant reason behind the endurance failure. The HRS retention was found to degrade with the switching cycle, whereas the LRS retention was improved. These cycle-dependent retention characteristics strongly supported the Cu accumulation hypothesis. By implicatively tailoring the proper pulse condition, more than 10^9^ cycles has been achieved. This comprehensive understanding of the failure mechanisms in this oxide-based ECM device will provide a valuable guideline for improving cell and array performance.

## Methods

### Hybrid integration of 1T1R array

The CMOS transistor was fabricated using a standard 0.13 μm logic process and RRAM devices with Cu/HfO_x_/Pt structures integrated onto it. A chemical mechanical polished Cu plug served as the bottom electrode. The HfO_x_ switching layer and Pt top electrode were grown by ion beam sputtering and electron-beam evaporation successively, with respective thicknesses of 4 nm and 70 nm. The size of the RRAM device was about 300 nm × 400 nm, defined by the bottom Cu plug. The memory cells and bit lines were patterned by a liftoff process. The bit lines were connected with the interfaces of the decoder to address the memory cells in the array individually. The channel width/length of the select transistor was 10 μm/1 μm.

### Characterization

The 1 kb ECM array was characterized by a self-made test system with a probe card in a semi-automatic probe station (Cascade Summit 12000). The DC and pulse endurance of the ECM cell were tested by a Keithely 4200 SCS semiconductor parameter analyzer connected to the experimental device. The current during the SET transition was limited by properly biasing the gate voltage on the selector transistor. The data retention of the HRS and LRS were performed in an oven of MEMMERT VO200. The resistance states of the programed devices in array were periodically checked with a read voltage of 0.1 V when cooled to room temperature. The TEM specimen was prepared by Focused ion beam (FIB). cutting and milling and the material compositions were analyzed by EDS and EELS.

## Author Contributions

H.L., X.X. and M.L. designed this work. X.X. and H.L. designed and fabricated the devices. H. L. and R.L. carried out the endurance measurement. X.X. performed the retention measurement. H.L. implemented the TEM analysis and interpreted the experiment data. Q.L., W.B., H.S., S.L. and L.L. contributed to the manuscript preparation. All of the authors discussed the experiments. M.L. and H.L. coordinated and supervised the research.

## Supplementary Material

Supplementary Information Supplementary Information
