# Supplementary material for: Evolution of conductive filament and its impact on reliability issues in oxide-electrolyte based resistive random access memory
Source: Sci Rep. 2015 Jan 14;5:7764. doi: 10.1038/srep07764 (PMC4293596; doi:10.1038/srep07764)
Supplement: Supplementary Information [file srep07764-s1.pdf]

Supplementary Information

Evolution of conductive filament and its impact on reliability issues in oxide-electrolyte based resistive random access memory

Hangbing Lv[†], Xiaoxin Xu[†], Hongtao Liu, Ruoyu Liu, Qi Liu, Writam Banerjee, Haitao Sun,

Shibing Long, Ling Li & Ming Liu

Laboratory of Nano-fabrication and Novel Devices Integrated Technology, Institute of Microelectronics, Chinese Academy of Sciences, Beijing 100029, People's Republic of China.

[†]These authors contributed equally to this work.

Correspondence and requests for materials should be addressed to M.L. (email: liuming@ime.ac.cn)

Table of Contents:

I. Integration of HfO_2 based ECM device on CMOS platform

II. The evolution of SET and RESET voltage with switching cycle

III. The evolution of tunnel gap length corresponding to the HRS degradation

I. Integration of HfO_2 based ECM device on CMOS platform.

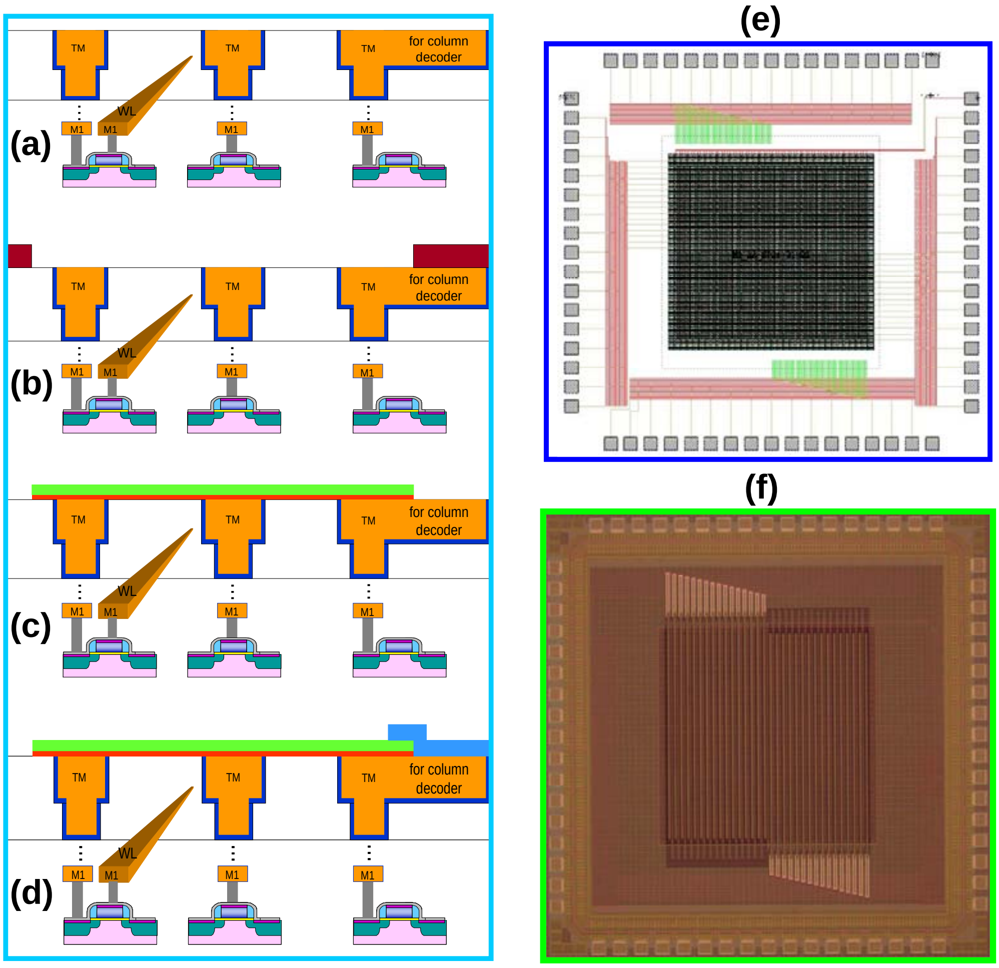

Figure S1. Hybrid integration of HfO_2 based ECM device on CMOS process. (a) The schematic of the cross-section of CMOS circuit after the CMP process of top metal (TM) formation. (b) Pattern the memory region and bit line by lithography. (c) Deposit the HfO_2 material and top electrode material. After a liftoff process, the ECM cells and parallel arranged BLs are formed. (d) Connect the BLs with the interfaces of decoder. (e) The layout of 1 kb RRAM array. (f) The optical image of 1kb 1T1R ECM array after hybrid integration.

II. The evolution of SET and RESET voltage with switching cycle

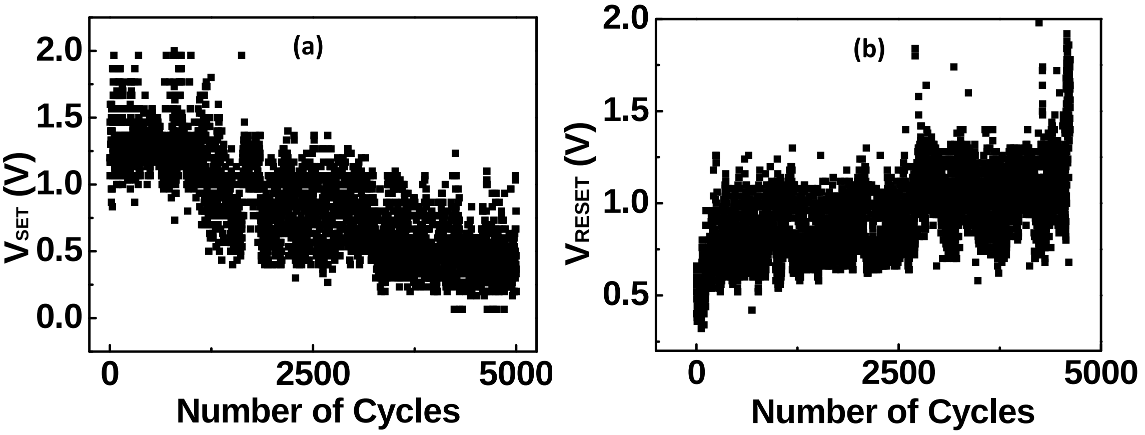

Figure S2. The relation of (a) V_{SET} and (b) V_{RESET} voltage with switching cycle. The V_{SET} is decreased with cycle. The V_{RESET} is increased with cycle. Although there exist some fluctuations for the cycle to cycle operation, the evolution trends for V_{SET} and V_{RESET} are clear.

III. The evolution of tunnel gap length corresponding to the HRS degradation

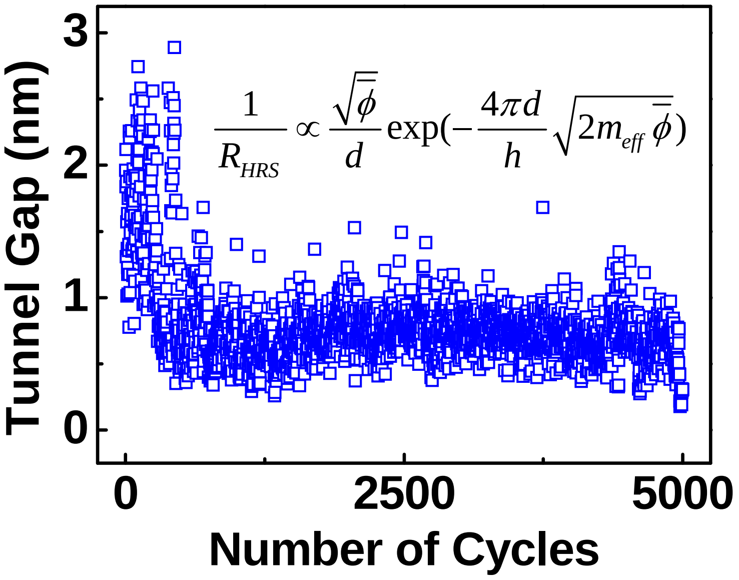

Figure S3. The evolution of tunnel gap length corresponding to the HRS change with cycle. Since the read voltage of resistance states is as low as 0.1 V, the HRS resistance can be modeled by low voltage

direct tunneling equation, as shown in the insert of figure, where $\bar{\phi}$ is the barrier height for electron tunneling, d is the gap length or the barrier width, h is the Planck constant, m_{eff} is effective electron mass. Assuming the electron tunneling barrier to be 2.0 eV and the filament with a size of 10 nm, the HRS after each switching cycle can be mirrored by the varied tunnel gap length. Although the calculated results may be deviated from the actual spatial gap due to the image force or different modeling parameters, it provides a guide of eye for gap length change with cycling.
